# Association of Preoperative Opioid Use With Mortality and Short-term Safety Outcomes After Total Knee Replacement

**DOI:** 10.1001/jamanetworkopen.2019.8061

**Published:** 2019-07-31

**Authors:** Seoyoung C. Kim, Yinzhu Jin, Yvonne C. Lee, Joyce Lii, Patricia D. Franklin, Daniel H. Solomon, Jessica M. Franklin, Jeffrey N. Katz, Rishi J. Desai

**Affiliations:** 1 Division of Pharmacoepidemiology and Pharmacoeconomics, Brigham and Women’s Hospital, Harvard Medical School, Boston, Massachusetts; 2 Division of Rheumatology, Immunology and Allergy, Brigham and Women’s Hospital, Harvard Medical School, Boston, Massachusetts; 3 Division of Rheumatology, Northwestern University, Chicago, Illinois; 4 Department of Medical Social Sciences, Northwestern University, Chicago, Illinois; 5 Department of Orthopedic Surgery, Brigham and Women’s Hospital, Harvard Medical School, Boston, Massachusetts

## Abstract

**Importance:**

Prescription opioid use is common among patients with moderate to severe knee osteoarthritis before undergoing total knee replacement (TKR). Preoperative opioid use may be associated with worse clinical and safety outcomes after TKR.

**Objective:**

To determine the association of preoperative opioid use among patients 65 years and older with mortality and other complications at 30 days post-TKR.

**Design, Setting, And Participants:**

This cohort study used claims data from January 1, 2010, to December 31, 2014, from a random sample of US Medicare enrollees 65 years and older who underwent TKR. Based on opioid dispensing in 360 days prior to TKR, patients were classified as continuous (≥1 opioid dispensing in each of the past 12 months) or intermittent (any dispensing of opioids in the past 12 months but not continuous use) opioid users or as opioid-naive patients (no opioids dispensed in the past 12 months). Data analyses were conducted from October 3, 2017, to November 8, 2018.

**Main Outcomes and Measures:**

Primary outcomes included in-hospital mortality and 30-day post-TKR mortality, hospital readmission, and revision operation. Secondary safety outcomes at 30 days post-TKR included opioid overdose and vertebral and nonvertebral fracture. Multivariable Cox proportional hazards models estimated hazard ratios (HRs) and 95% CIs.

**Results:**

Of 316 593 patients (mean [SD] age, 73.9 [5.8] years; 214 677 [67.8%] women) who underwent TKR, 22 895 (7.2%) were continuous opioid users, 161 511 (51.0%) were intermittent opioid users, and 132 187 (41.7%) were opioid naive. In-hospital mortality occurred in 276 patients (0.09%). At 30 days post-TKR, 828 patients (0.26%) died, 16 786 patients (5.30%) had hospital readmission, and 921 patients (0.29%) had a revision operation. All primary and secondary outcomes occurred more frequently among continuous opioid users compared with opioid-naive patients. Compared with opioid-naive patients and after adjusting for demographic characteristics, combined comorbidity score, number of different prescription medications, and frailty, continuous opioid users had greater risk of revision operations (HR, 1.63; 95% CI, 1.15-2.32), vertebral fractures (HR, 2.37; 95% CI, 1.37-4.09), and opioid overdose (HR, 4.82; 95% CI, 1.36-17.07) at 30 days post-TKR. However, after adjusting covariates, there were no statistically significant differences in in-hospital (HR, 1.18; 95% CI, 0.73-1.90) or 30-day (HR, 1.05; 95% CI, 0.73-1.51) mortality between continuous opioid users and opioid-naive patients.

**Conclusions and Relevance:**

After adjusting for baseline risk profiles, including comorbidities and frailty, continuous opioid users had a higher risk of revision operations, vertebral fractures, and opioid overdose at 30 days post-TKR but not of in-hospital or 30-day mortality, compared with opioid-naive patients. These results highlight the need for better understanding of patient characteristics associated with chronic opioid use to optimize preoperative assessment of overall risk after TKR.

## Key Points

**Question:**

Is preoperative opioid use associated with mortality and short-term safety outcomes after total knee replacement (TKR) surgical procedures?

**Findings:**

In this cohort study of 316 593 patients 65 years and older who underwent a TKR, 22 895 patients (7.2%) had a history of continuous opioids use in 360 days prior to surgery. After adjusting for the baseline risk profile, continuous opioid users had higher risks of revision operations, vertebral fractures, and opioid overdose at 30 days post-TKR but not of in-hospital or 30-day mortality, compared with opioid-naive patients.

**Meaning:**

Better understanding of patient characteristics associated with chronic opioid use is needed to optimize preoperative assessment of overall risk after TKR.

## Teaser

This cohort study examines the association of preoperative opioid use with mortality and safety outcomes after total knee replacement in patients 65 years and older.

## Introduction

Overuse of prescription opioids in the United States has been a threat to public health during the past decade as opioid analgesic sales increased 4-fold from 1999 to 2010.^1^ While the use of opioids is prevalent across all adult age groups, adults older than 60 years use prescription opioids at a rate almost 2-fold more than younger adults aged 20 to 39 years.^1^ In older patients, given the known cardiovascular risks of nonsteroidal anti-inflammatory drugs (NSAIDs), the threshold for using opioids has decreased; opioids are used increasingly among elderly individuals and people with cardiovascular risk factors.^2^

Given increasing concern about opioid overuse and subsequent restrictions on opioid prescribing, management of chronic painful conditions, such as osteoarthritis (OA), has become particularly challenging. Opioid analgesics are often prescribed to relieve pain in patients with moderate to severe symptomatic OA not responsive to NSAIDs or acetaminophen. Based on the data from the US Medicare Current Beneficiary Survey,^2^ more than 40% of patients with OA with a mean age of 77 years received an opioid prescription in 2009. A 2017 study^3^ among a US commercially insured population of patients undergoing hip or knee arthroplasty found that 87.1% had received at least 1 dispensing for opioids in the year prior to the surgical procedure. A Medicare-based cohort study^4^ using data from 2010 through 2014 found that 42.3% of older patients with OA used prescription opioids for less than 90 days and 16.5% of older patients used prescription opioids for longer than 90 days in the year prior to total joint replacement.

Several studies have raised concerns about potential associations of opioid use prior to total joint replacement with postsurgical adverse outcomes, including persistent pain, stiffness, patient satisfaction, and requirement of additional surgical procedures.^5,6,7,8^ Furthermore, in 2 studies of patients with a mean age of 80 years with arthritis, compared with nonselective NSAIDs, patients who used opioids had a 5-fold increased risk of fracture^9^ and a 1.9-fold increased risk of cardiovascular events and death.^10^ However, to our knowledge, limited information is available on the association of preoperative opioid use with a broad range of post–total knee replacement (TKR) outcomes after accounting for patients’ preoperative risk profile among a nationally representative cohort of patients. Therefore, we sought to determine the association of preoperative opioid use with short-term safety outcomes after TKR, including in-hospital mortality and mortality, TKR complications, and safety events at 30 days post-TKR among Medicare enrollees in the United States. We also assessed these outcomes at 60 and 90 days after TKR.

## Methods

### Data Source

We used claims data from Medicare Parts A (inpatient services), B (outpatient services), and D (pharmacy claims) from January 1, 2010, to December 31, 2014. Medicare is a federally funded program that provides health care coverage for nearly all legal residents of the United States older than 65 years and some individuals with disabilities younger than 65 years. This database contains longitudinal information on Medicare enrollees’ medical diagnoses recorded with the *International Classification of Diseases, Ninth Revision, Clinical Modification* (*ICD-9-CM*)^11^ codes, medical procedures recorded as *Current Procedural Terminology* or *ICD-9-CM* procedure codes, and medication dispensing recorded using National Drug Codes. The protocol was reviewed and approved by the Institutional Review Board of the Brigham and Women’s Hospital, which granted a waiver of informed consent, as this study exclusively used deidentified patient data. The data use agreement was in place with the US Centers for Medicare & Medicaid Services. The reporting of this study is in accordance with the Strengthening the Reporting of Observational Studies in Epidemiology (STROBE) reporting guideline.

### Study Cohort

We obtained a random sample of 1 million patients who underwent a total knee or hip replacement from January 1, 2010, to December 31, 2014. We then selected patients with continuous enrollment in Medicare Parts A, B, and D for at least 360 days prior to TKR. All patients were required to have a diagnosis of OA or rheumatoid arthritis and be 65 years or older at the time of the index TKR (ie, index date). We excluded patients who had no claims during the 360-day baseline period (ie, those who were Medicare eligible but may have been receiving care through alternate health insurance coverage) or those who had both TKR and total hip replacement performed on the same date. Patients were included in the cohort once at the time of their first TKR, even if they had multiple eligible TKR dates identified during the study.

### Preoperative Opioid Use Pattern

We identified opioids based on 16 different generic names, including buprenorphine, codeine, dihydrocodeine, fentanyl, hydrocodone, hydromorphone, levorphanol, meperidine, methadone, morphine, oxycodone, oxymorphone, pentazocine, propoxyphene, tapentadol, and tramadol. Based on dispensing of opioids during the 360-day baseline period prior to TKR, patients were classified as (1) continuous opioid users (ie, ≥1 dispensing in each of twelve 30-day blocks prior to TKR), (2) intermittent opioid users (ie, any dispensing of opioids but not continuous use), or (3) opioid naive (ie, no opioid dispensing in the past 12 months).

### Outcomes of Interest

The primary outcomes of interest were (1) in-hospital mortality (ie, death during the hospitalization for TKR), (2) 30-day mortality, (3) 30-day hospital readmission of any kind, and (4) 30-day TKR revision operations. Based on previously published algorithms using diagnosis or procedure codes, we assessed the following secondary safety outcomes at 30 days post-TKR: (1) opioid overdose^12,13^; (2) a composite cardiovascular endpoint, including myocardial infarction and stroke^14,15^; (3) nonvertebral fracture^16^; (4) vertebral fracture^17^; (5) respiratory distress^18^; (6) pneumonia^19^; and (7) bowel obstruction.^20^ In addition, we examined the rate of primary and secondary outcomes at 60 and 90 days post-TKR as sensitivity analyses.

### Covariates

During the 360-day baseline period prior to TKR, we assessed patient demographic characteristics (ie, age, sex, race/ethnicity [self-reported in the Medicare enrollment database], and region of residence), comorbidities (eg, falls, migraine, neuropathic pain, back pain, fractures, hyperlipidemia, hypertension, atrial fibrillation, heart failure, coronary heart disease, stroke, chronic kidney disease, diabetes, obesity, malignant tumors, smoking, substance use disorder, osteoporosis, psychosis, depression, sleep disorder, and anxiety), medication use (ie, NSAIDs, selective cyclooxygenase 2 inhibitors, corticosteroids, anticonvulsants, antidepressants, antipsychotics, benzodiazepines, other anxiolytics, and total number of unique prescriptions by generic name), and health care utilization patterns. These covariates were defined using *ICD-9-CM* diagnosis or procedure codes, *Current Procedural Terminology* codes, or National Drug Codes. In addition, to better assess older patients’ health status and physical function, we estimated a combined comorbidity score^21^ that incorporated 20 different medical conditions, including heart failure, renal failure, respiratory disease, cirrhosis, and malignant tumors, and a claims-based frailty index.^22^ Based on the frailty index score, patients were categorized into 4 groups: robust (<0.15), prefrail (0.15-0.24), mildly frail (0.25-0.34), and moderately to severely frail (>0.34).^23^

### Statistical Analysis

We cross-tabulated baseline characteristics of patients by preoperative opioid use patterns. We calculated the proportion of patients who experienced primary or secondary outcomes of interest during 30 days post-TKR. Separate crude Cox proportional hazards models estimated hazard ratios (HRs) and 95% CIs for primary and secondary outcomes. To adjust for confounding, we performed partial adjustment for demographic factors only (model 1) and full adjustment for demographic characteristics, combined comorbidity score, frailty, and number of prescription drugs (model 2). We also repeated these steps for 60 and 90 days of follow-up after the surgical procedure. In addition, we performed a sensitivity analysis after excluding patients with malignant tumors to focus exclusively on patients who received opioids for chronic noncancer pain. All analyses were conducted in SAS statistical software version 9.4 (SAS Institute).

## Results

### Study Patients

After applying the inclusion and exclusion criteria, the final study cohort included 316 593 patients who underwent TKR (mean [SD] age, 73.9 [5.8] years; 214 677 [67.8%] women) (Figure). Of these patients, 184 406 (58.2%) had any use of opioids in the 360 days prior to TKR, including 22 895 continuous opioid users (7.2%) and 161 511 intermittent opioid users (51.0%); 132 187 patients (41.7%) were opioid naive prior to the surgical procedure. The mean (SD) ages were 72.7 (5.7) years among continuous opioid users, 73.7 (5.7) years among intermittent opioid users, and 74.3 (5.8) years among opioid-naive patients. Continuous opioid users were more likely to be women and black and to live in the South. Continuous opioid users had more comorbidities, including diabetes, obesity, back pain, malignant tumors, cardiovascular disease, sleep disorder, psychiatric disorders, and substance use disorder. Furthermore, continuous opioid users were more frail than opioid-naive patients. Use of other analgesic medications, benzodiazepines, and anticonvulsants was more frequently seen among continuous opioid users than opioid-naive patients. Table 1 summarizes preoperative characteristics of the study population. A total of 60 040 patients (19.0%) had a history of malignant tumors. In the subgroup of 256 553 patients with no baseline malignant tumors, 148 926 (58.0%) had any use of opioids in 360 days pre-TKR and 190 241 (7.5%) were continuous opioid users (Table 1).

**Figure.**
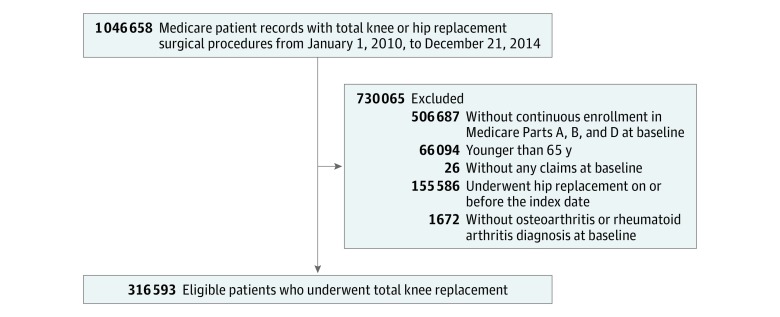
Cohort Selection Flow

**Table 1.** Patient Characteristics in 360 Days Prior to Total Knee Replacement

Characteristic	No. (%)		
	Opioid Users		Opioid-Naive Patients (n = 132 187)
	Continuous (n = 22 895)	Intermittent (n = 161 511)	
Age, mean (SD), y	72.7 (5.7)	73.7 (5.7)	74.3 (5.8)
Women	17 432 (76.1)	112 574 (69.7)	84 671 (64.1)
Race/ethnicity			
White	20 227 (88.3)	144 455 (89.4)	121 605 (92.0)
Black	1863 (8.1)	8967 (5.6)	4536 (3.4)
Hispanic	307 (1.3)	3336 (2.1)	1730 (1.3)
Other	309 (1.3)	2664 (1.6)	2280 (1.7)
Region			
Northeast	2401 (10.5)	22 443 (13.9)	24 633 (18.6)
Midwest	5905 (25.8)	43 364 (26.8)	41 153 (31.1)
South	10 350 (45.2)	65 244 (40.4)	44 550 (33.7)
West	4231 (18.5)	30 158 (18.7)	21 591 (16.3)
Comorbidities			
Hypertension	20 537 (89.7)	139 063 (86.1)	107 306 (81.2)
Diabetes	8737 (38.2)	54 213 (33.6)	37 498 (28.4)
Obesity	5199 (22.7)	30 791 (19.1)	18 210 (13.8)
Back pain	16 303 (71.2)	85 228 (52.8)	46 908 (35.5)
Neuropathic pain	10 295 (45.0)	52 270 (32.4)	24 768 (18.7)
Malignant tumor	3654 (16.0)	31 826 (19.7)	24 560 (18.6)
Coronary heart disease	2579 (11.3)	14 478 (9.0)	8499 (6.4)
Chronic kidney disease	3675 (16.1)	19 636 (12.2)	10 883 (8.2)
Heart failure	3511 (15.3)	16 247 (10.1)	8389 (6.3)
Hip fracture	122 (0.5)	682 (0.4)	232 (0.2)
Migraine	3604 (15.7)	17 773 (11.0)	8354 (6.3)
Sleep disorder	6219 (27.2)	32 891 (20.4)	17 777 (13.4)
Depression	7167 (31.3)	28 744 (17.8)	13 204 (10.0)
Anxiety disorder	5414 (23.6)	20 973 (13.0)	9958 (7.5)
Bipolar disorder	594 (2.6)	2058 (1.3)	930 (0.7)
Substance use disorder	291 (1.3)	343 (0.2)	57 (0)
Alcohol use disorder	365 (1.6)	1615 (1.0)	771 (0.6)
Tobacco use	4900 (21.4)	23 465 (14.5)	12 283 (9.3)
Rheumatoid arthritis	2442 (10.7)	9831 (6.1)	4318 (3.3)
Frailty^a^			
Robust	3502 (15.3)	52 549 (32.5)	65 442 (49.5)
Prefrail	14 969 (65.4)	96 700 (59.9)	63 689 (48.2)
Mild frailty	4049 (17.7)	11 338 (7.0)	2924 (2.2)
Moderate to severe frailty	375 (1.6)	924 (0.6)	132 (0.1)
Combined comorbidity score, mean (SD)	1.9 (2.6)	1.3 (2.3)	0.8 (1.8)
Medication use			
NSAIDs	10 584 (46.2)	71 998 (44.6)	39 018 (29.5)
Cyclooxygenase 2 inhibitors	2773 (12.1)	18 651 (11.5)	9902 (7.5)
Oral corticosteroids	10 527 (46.0)	64 672 (40.0)	37 443 (28.3)
Antidepressants	12 371 (54.0)	53 669 (33.2)	26 321 (19.9)
Benzodiazepines	5117 (22.3)	18 913 (11.7)	8240 (6.2)
Bisphosphonates	1823 (8.0)	12 256 (7.6)	8787 (6.6)
Anticonvulsants	9127 (39.9)	32 130 (19.9)	11 733 (8.9)
Health care utilization, mean (SD)			
No. of unique prescription drugs	15.5 (6.7)	12.2 (5.6)	7.8 (4.5)
No. of emergency department visits	0.8 (1.7)	0.5 (1.1)	0.2 (0.6)
No. of visits to any physician	16.2 (9.6)	13.9 (8.0)	10.7 (6.5)
No. of acute hospitalizations	0.4 (0.8)	0.3 (0.6)	0.1 (0.4

Abbreviation: NSAIDs, nonsteroidal anti-inflammatory drugs.

^a^ Based on a frailty index score, patients were categorized into 4 groups, robust (<0.15), prefrail (0.15-0.24), mildly frail (0.25-0.34), and moderately to severely frail (>0.34).

### Primary Outcomes

Among the full cohort, in-hospital mortality occurred in 282 patients (0.09%). At 30 days post-TKR, 828 patients (0.26%) died, 16 786 patients (5.30%) had hospital readmission, and 921 patients (0.29%) had a revision operation. In-hospital mortality occurred in 27 continuous opioid users (0.12%), 165 intermittent opioid users (0.10%), and 84 opioid-naive patients (0.06%) (Table 2). The all-cause mortality rate was higher among continuous opioid users compared with intermittent opioid users or opioid-naive patients at 30 days (75 continuous opioid users [0.33%]; 451 intermittent opioid users [0.28%]; 302 opioid-naive patients [0.23%]), 60 days (123 continuous opioid users [0.54%]; 628 intermittent opioid users [0.39%]; 412 opioid-naive patients [0.31%]), and 90 days (156 continuous opioid users [0.68%]; 760 intermittent opioid users [0.47%]; 499 opioid-naive patients [0.38%]) after TKR. Hospital readmission at 30 days post-TKR occurred in 1672 continuous opioid users (7.30%), 9027 intermittent opioid users (5.59%), and 6087 opioid-naive patients (4.60%). Revision operations within 30 days post-TKR were generally infrequent but noted in 112 continuous opioid users (0.49%), 524 intermittent opioid users (0.32%), and 285 opioid-naive patients (0.22%). Additionally, at 60 and 90 days post-TKR, all primary outcomes occurred more frequently in continuous opioid users vs opioid naive patients and in intermittent opioid users vs opioid-naive patients (Table 2). As summarized in Table 3, the unadjusted HR among continuous opioid users vs opioid-naive patients was greater for in-hospital mortality (HR, 1.95; 95% CI, 1.25-3.03), 30-day mortality (HR, 1.52; 95% CI, 1.09-2.11), 30-day hospital readmission (HR, 1.47; 95% CI, 1.36-1.60), and 30-day revision operation (HR, 2.55; 95% CI; 1.86-3.48). In the partially adjusted model 1, the HR remained greater for continuous opioid uses for these primary outcomes compared with opioid-naive patients (Table 3). In the fully adjusted model 2, continuous opioid users vs opioid-naive patients were no longer associated with in-hospital mortality (HR, 1.18; 95% CI, 0.73-1.90), 30-day mortality (HR, 1.05; 95% CI, 0.73-1.51), or 30-day hospital readmission (HR, 1.06; 95% CI, 0.97-1.16) after TKR (Table 3). However, continuous opioid use was associated with a greater risk of a revision operation (HR, 1.63; 95% CI, 1.15-2.32) at 30 days post-TKR.

**Table 2.** All-Cause Mortality and Short-term Complications After Total Knee Replacement Stratified by Preoperative Opioid Use Patterns

Outcome	Events, No. (%)		
	Opioid Users		Opioid-Naive Patients (n = 132 187)
	Continuous (n = 22 895)	Intermittent (n = 161 511)	
All-cause mortality			
In hospital	27 (0.12)	165 (0.10)	84 (0.06)
30 d	75 (0.33)	451 (0.28)	302 (0.23)
60 d	123 (0.54)	628 (0.39)	412 (0.31)
90 d	156 (0.68)	760 (0.47)	499 (0.38)
Hospital readmission			
30 d	1672 (7.30)	9027 (5.59)	6087 (4.60)
60 d	2545 (11.12)	13 305 (8.24)	8891 (6.73)
90 d	3296 (14.40)	16 915 (10.47)	11 228 (8.49)
Revision operation			
30 d	112 (0.49)	524 (0.32)	285 (0.22)
60 d	162 (0.71)	778 (0.48)	424 (0.32)
90 d	198 (0.86)	912 (0.56)	503 (0.38)

**Table 3.** All-Cause Mortality, Short-term Complications, and Safety Outcomes After Total Knee Replacement Among Continuous Opioid Users vs Opioid-Naive Patients

Outcome	Hazard Ratio (95% CI)		
	Unadjusted	Model 1^a^	Model 2^b^
All-cause mortality			
In hospital	1.95 (1.25-3.03)	2.36 (1.51-3.69)	1.18 (0.73-1.90)
30 d	1.52 (1.09-2.11)	1.88 (1.34-2.63)	1.05 (0.73-1.51)
60 d	1.67 (1.24-2.23)	2.07 (1.55-2.79)	1.12 (0.82-1.54)
90 d	1.50 (1.14-1.99)	1.87 (1.41-2.49)	0.99 (0.73-1.35)
Hospital readmission			
30 d	1.47 (1.36-1.60)	1.57 (1.45-1.71)	1.06 (0.97-1.16)
60 d	1.57 (1.47-1.67)	1.67 (1.56-1.78)	1.12 (1.04-1.20)
90 d	1.63 (1.54-1.73)	1.74 (1.64-1.84)	1.19 (1.12-1.27)
Revision operation			
30 d	2.55 (1.86-3.48)	2.59 (1.88-3.57)	1.63 (1.15-2.32)
60 d	2.21 (1.70-2.87)	2.31 (1.77-3.01)	1.40 (1.05-1.88)
90 d	2.37 (1.87-2.99)	2.47 (1.94-3.14)	1.58 (1.21-2.05)
Opioid overdose			
30 d	8.89 (2.82-28.00)	8.50 (2.67-27.12)	4.82 (1.36-17.07)
60 d	15.41 (5.43-43.76)	15.1 (5.26-43.34)	7.91 (2.50-25.02)
90 d	25.99 (9.75-69.25)	26.6 (9.89-71.57)	13.64 (4.70-39.55)
Nonvertebral fracture			
30 d	3.08 (1.26-7.56)	3.00 (1.21-7.42)	1.89 (0.69-5.13)
60 d	2.24 (1.25-4.02)	2.23 (1.24-4.02)	1.50 (0.78-2.86)
90 d	2.44 (1.62-3.68)	2.57 (1.70-3.90)	1.75 (1.11-2.77)
Vertebral fracture			
30 d	4.40 (2.70-7.14)	4.69 (2.85-7.73)	2.37 (1.37-4.09)
60 d	4.22 (3.10-5.75)	4.51 (3.29-6.19)	2.42 (1.70-3.44)
90 d	4.18 (3.26-5.35)	4.41 (3.43-5.68)	2.36 (1.79-3.13)
Myocardial infarction or stroke			
30 d	1.02 (0.63-1.65)	1.21 (0.74-1.97)	0.76 (0.45-1.27)
60 d	1.11 (0.75-1.66)	1.34 (0.89-2.00)	0.85 (0.55-1.31)
90 d	1.25 (0.88-1.77)	1.50 (1.05-2.14)	0.90 (0.62-1.32)
Respiratory distress			
30 d	2.13 (0.77-5.85)	2.17 (0.78-6.01)	1.04 (0.35-3.14)
60 d	2.66 (1.22-5.78)	2.72 (1.24-5.97)	1.21 (0.51-2.85)
90 d	2.58 (1.36-4.91)	2.62 (1.37-5.01)	1.21 (0.59-2.50)
Pneumonia			
30 d	2.04 (1.32-3.14)	2.31 (1.49-3.57)	1.10 (0.68-1.80)
60 d	2.02 (1.39-2.93)	2.26 (1.55-3.30)	1.06 (0.69-1.61)
90 d	2.29 (1.63-3.20)	2.55 (1.82-3.59)	1.15 (0.79-1.68)
Bowel obstruction			
30 d	1.84 (1.04-3.27)	2.04 (1.14-3.65)	1.40 (0.74-2.63)
60 d	1.87 (1.15-3.04)	2.06 (1.26-3.36)	1.38 (0.81-2.35)
90 d	1.86 (1.20-2.87)	2.02 (1.30-3.13)	1.36 (0.84-2.19)

^a^ Adjusted for age, sex, race/ethnicity, and region of residence.

^b^ Adjusted for age, sex, race/ethnicity, region of residence, combined comorbidity score, frailty score, and number of unique prescription drugs.

### Secondary Safety Outcomes

Table 4 presents the results from the secondary safety outcome analysis. Opioid overdose occurred infrequently after TKR across the 3 groups. At 30 days post-TKR, 11 continuous opioid users (0.05%) experienced an opioid overdose, compared with 41 intermittent opioid users (0.03%) and fewer than 11 opioid-naive patients (<0.01%) (as required by the data use agreement with the Centers for Medicare & Medicaid Services, actual numbers for counts less than 11 are suppressed). The secondary outcomes at 30, 60, and 90 days post-TKR were generally more common among continuous opioid users than opioid-naive patients. Similarly, the unadjusted HR among continuous opioid users was greater for opioid overdose (HR, 8.89; 95% CI, 2.82-28.00), nonvertebral fractures (HR, 3.08; 95% CI, 1.26-7.56), vertebral fractures (HR, 4.40; 95% CI, 2.70-7.14), pneumonia (HR, 2.04; 95% CI, 1.32-3.14), and bowel obstruction (HR, 1.84; 95% CI, 1.04-3.27). After partial adjustment (model 1) for demographic factors, the HR remained higher for continuous opioid users compared with opioid-naive patients for opioid overdose (HR, 8.50; 95% CI, 2.67-27.12), nonvertebral fractures (HR, 3.00; 95% CI, 1.21-7.42), vertebral fractures (HR, 4.69; 95% CI, 2.85-7.73), pneumonia (HR, 2.31; 95% CI, 1.49-3.57), and bowel obstruction (HR, 2.04; 95% CI, 1.14-3.65) at 30 days post-TKR (Table 3). In the fully adjusted model 2 (Table 3), continuous opioid use was only associated with a greater risk of opioid overdose (HR, 4.82; 95% CI, 1.36-17.07) and vertebral fractures (HR, 2.37; 95% CI, 1.37-4.09) at 30 days post-TKR. Similar patterns were seen in the analyses for the outcomes at 60 and 90 days post-TKR.

**Table 4.** Short-term Safety Outcomes After Total Knee Replacement Stratified by Preoperative Opioid Use Patterns

Outcome	Events, No. (%)		
	Opioid Users		Opioid-Naive Patients (n = 132 187)
	Continuous (n = 22 895)	Intermittent (n = 161 511)	
Opioid overdose			
30 d	11 (0.05)	41 (0.03)	<11 (<0.01)^a^
60 d	23 (0.10)	49 (0.03)	11 (0.01)
90 d	34 (0.15)	53 (0.03)	11 (0.01)
Nonvertebral fracture			
30 d	17 (0.07)	87 (0.05)	45 (0.03)
60 d	48 (0.21)	210 (0.13)	110 (0.08)
90 d	87 (0.38)	353 (0.22)	195 (0.15)
Vertebral fracture			
30 d	57 (0.25)	235 (0.15)	95 (0.07)
60 d	129 (0.56)	504 (0.31)	225 (0.17)
90 d	205 (0.90)	767 (0.47)	347 (0.26)
Myocardial infarction or stroke			
30 d	43 (0.19)	288 (0.18)	237 (0.18)
60 d	65 (0.28)	407 (0.25)	321 (0.24)
90 d	86 (0.38)	518 (0.32)	396 (0.30)
Respiratory distress			
30 d	15 (0.07)	65 (0.04)	34 (0.03)
60 d	29 (0.13)	91 (0.06)	53 (0.04)
90 d	41 (0.18)	106 (0.07)	65 (0.05)
Pneumonia			
30 d	59 (0.26)	258 (0.16)	156 (0.12)
60 d	89 (0.39)	368 (0.23)	218 (0.16)
90 d	124 (0.54)	469 (0.29)	258 (0.20)
Bowel obstruction			
30 d	35 (0.15)	159 (0.10)	118 (0.09)
60 d	49 (0.21)	224 (0.14)	157 (0.12)
90 d	58 (0.25)	281 (0.17)	188 (0.14)

^a^ As required by the data use agreement with the Centers for Medicare & Medicaid Services, actual numbers for counts less than 11 are suppressed.

Using the fully adjusted model 2, compared with opioid-naive patients, intermittent opioid users were not associated with increased in-hospital mortality (HR, 1.20; 95% CI, 0.90-1.59), 30-day mortality (HR, 1.10; 95% CI, 0.90-1.34), or 30-day hospital readmission (HR, 0.99; 95% CI, 0.94-1.04). The fully adjusted HR associated with intermittent opioid users compared with opioid-naive patients was 1.29 (95% CI, 1.04-1.61) for revision operations, 3.07 (95% CI, 1.12-8.40) for opioid overdose, and 1.54 (95% CI, 1.04-2.28) for vertebral fractures at 30 days post-TKR (eTable 1 in the Supplement).

In the sensitivity analysis excluding 256 553 patients with malignant tumors (eTable 2 in the Supplement), we also found consistent results. In the fully adjusted model 2, continuous opioid users compared with opioid-naive patients were associated with a greater risk of revision operations (HR, 1.66; 95% CI, 1.15-2.40), opioid overdose (HR, 3.65; 95% CI, 0.98-13.66), and vertebral fracture (HR, 2.32; 95% CI, 1.28-4.21) at 30 days but not with risk of in-hospital mortality (HR, 0.98; 95% CI, 0.56-1.69), 30-day mortality (HR, 0.95; 95% CI, 0.63-1.43), or 30-day hospital readmission (HR, 1.05; 95% CI, 0.96-1.16) (eTable 3 in the Supplement).

## Discussion

In this large cohort of older Medicare enrollees with OA (mean age, >73 years), 58.3% had used opioids at least once in the year prior to TKR, and 7.2% had continuous opioid use, defined by a dispensing for opioid at least once every month for 12 months before the surgical procedure. Compared with opioid-naive patients, continuous opioid users had greater in-hospital mortality, all-cause mortality, revision operations, hospital readmission, and other safety events after TKR. After adjusting for differences in patient characteristics, we found no association of continuous preoperative opioid use with in-hospital mortality or with all-cause mortality, hospital readmission, myocardial infarction or stroke, or pneumonia at 30 days post-TKR (Table 3). However, in our fully adjusted analyses, continuous opioid use was associated with a higher risk of early (ie, 30-day) revision operation and vertebral fracture and of opioid overdose at 30, 60, and 90 days after TKR. Multivariable model 2 HRs for continuous opioid use vs no use were elevated for nonvertebral fractures, respiratory distress, and bowel obstruction after TKR, but the differences were not statistically significant. In the model 2 adjusted analyses, we found no association of continuous opioid use with in-hospital mortality, all-cause mortality, 30-day hospital readmission, myocardial infarction or stroke, or pneumonia post-TKR. Intermittent use of opioids vs no opioid use was also associated with an increased risk of revision operations, vertebral fractures, and opioid overdose at 30 days post-TKR. We found consistent results in a sensitivity analysis excluding patients with malignant tumors at baseline.

The key clinical question is whether long-term use of opioids itself is a risk factor for worse outcomes after a surgical procedure or if patients’ conditions that lead to long-term use of opioids are a risk factor. As seen in previous studies,^3,24,25^ the use of prescription opioids in these older patients was preoperatively prevalent in our study, regardless of baseline history of malignant tumors. Furthermore, a considerable number of patients had continuous use of opioids in the year prior to TKR, and these continuous opioid users had a higher rate of short-term complications after TKR compared with opioid-naive patients. In a small study by Zywiel et al^8^ of 98 patients who received TKR, patients who had preoperative long-term use of opioids had worse clinical outcomes and higher complication rates. Zywiel et al^8^ suggested alternative pain management with nonopioids. A 2018 study^24^ of more than 300 000 total joint replacements using claims data from a US commercial insurance database reported a greater risk of early revision operation and 30-day readmission among patients with longer than 60 days of preoperative opioid use vs those with no opioid use after adjusting for age, sex, and combined comorbidity score. Similar to these studies,^8,24^ we also noted a higher rate of revision operations and other safety events among continuous opioid users vs opioid-naive patients. It is uncommon to perform a revision operation during a postoperative period of 30, 60, or 90 days. Although, to our knowledge, the underlying mechanism associated with preoperative opioid use with early revision operation is not fully understood, it is important to note that continuous opioid users were more frail and had more comorbidities and other prescription drug use compared with opioid-naive patients. These patient characteristics might have been associated with more infection, persistent pain, or other unusual conditions, ultimately needing a revision operation. The HR of 1.95 (95% CI, 1.25-3.03) for in-hospital mortality associated with continuous opioid use in the unadjusted analysis was attenuated to 1.18 (95% CI, 0.73-1.90) in the multivariable model 2, which was adjusted for demographic factors, region, combined comorbidity score, frailty, and number of unique prescription drugs. Similarly, the unadjusted HR for pneumonia was 2.04 (95% CI, 1.32-3.14) for continuous opioid users vs opioid-naive patients, and it attenuated to 1.10 (95% CI, 0.68-1.80) in the adjusted model 2 analysis. Unlike a 2010 study^10^ that found an increased risk of cardiovascular events associated with opioid vs NSAID use, we found no cardiovascular risk associated with continuous opioid users vs opioid-naive patients.

These findings suggest that differences in the baseline risk profile between continuous opioid users and opioid-naive patients may contribute more to the observed higher rate of mortality and some of the short-term safety events than the pattern of preoperative opioid use itself. In other words, it may be not possible to reduce the rate of some of the short-term complications after TKR even if use of opioids is minimized. Nonetheless, observation from our study and previous studies^8,24^ suggest that, even if it is not a truly independent risk factor, preoperative long-term use of opioids may be a marker with an unfavorable risk profile leading to poor postoperative outcome. As such, evaluation of patients’ preoperative opioid use patterns may be helpful in planning a more rigorous monitoring strategy after a common elective surgical procedure, such as TKR.

### Strengths and Limitations

Strengths of this study include the large size of the study cohort and high generalizability, as Medicare covers all legal residents 65 years and older in the United States. We also conducted a comprehensive assessment of short-term surgical complications as well as various safety events directly or indirectly associated with opioid use. Furthermore, we conducted a thorough evaluation of patient characteristics prior to their surgical procedures and accounted for many important variables, including comorbidities and frailty, in the analyses. Lastly, we examined the complication and safety event rates at 30, 60, and 90 days post-TKR for a complete postoperative outcome evaluation.

This study has limitations. First, because we relied on diagnosis codes and pharmacy dispensing in Medicare data, there is a potential for misclassification of comorbidities or opioid use. We also do not have information on the reasons for opioid prescriptions. Second, because we evaluated short-term safety outcomes among patients who underwent an elective surgery (ie, TKR), rates of the outcomes were generally low, leading to imprecise estimates for some of the secondary outcomes, such as respiratory distress and bowel obstruction. Third, we did not have data on in-hospital opioid use or types of anesthesia during the index hospitalization, which may have had an important role in in-hospital mortality or some of the 30-day safety events, such as opioid overdose. Fourth, this observational study is subject to residual confounding among the groups.

## Conclusions

Among 316 593 older patients with knee arthritis enrolled in Medicare, preoperative use of prescription opioids was common: 58.3% of patients had at least 1 dispensing for opioids in 360 days prior to TKR. Compared with opioid-naive individuals, after adjusting for a baseline risk profile, including comorbidities and frailty, continuous preoperative opioid use was associated with a higher risk of revision operations, vertebral fractures, and opioid overdose at 30 days post-TKR but was no longer associated with in-hospital or 30-day mortality. Similarly, intermittent opioid use vs no opioid use was associated with a greater risk of revision operations, vertebral fractures, and opioid overdose at 30 days post-TKR, although to a lesser degree. It is important to recognize the harms of prescription opioids and minimize the doses or duration of opioids whenever possible. Nevertheless, our results suggest that differences in the baseline risk profile between opioid users and opioid-naive patients were likely more important contributing factors for in-hospital or short-term mortality, as well as some of the short-term safety events after TKR, than preoperative opioid use itself. Our study also highlights the need for better understanding of patient characteristics associated with chronic opioid use to optimize preoperative assessment of overall risk after TKR among older patients with arthritis.

## Notes

Supplement. **eTable 1.** All-cause Mortality and Short-term Safety Outcomes After Total Knee Replacement Among Intermittent Opioid Users vs Opioid-Naive Patients **eTable 2.** Patient Characteristics Prior to Total Knee Replacement Excluding Patients With Malignant Tumors **eTable 3.** All-cause Mortality and Short-term Safety Outcomes After Total Knee Replacement Among Continuous Opioid Users vs Opioid-Naive Patients Excluding Patients With Malignant Tumors
