# Supplementary material for: Association of Preoperative Opioid Use With Mortality and Short-term Safety Outcomes After Total Knee Replacement
Source: JAMA Netw Open. 2019 Jul 31;2(7):e198061. doi: 10.1001/jamanetworkopen.2019.8061 (PMC6669774; doi:10.1001/jamanetworkopen.2019.8061)
Supplement: Supplement. — eTable 1. All-cause Mortality and Short-term Safety Outcomes After Total Knee Replacement Among Intermittent Opioid Users vs Opioid-Naive Patients eTable 2. Patient Characteristics Prior to Total Knee Replacement Excluding Patients With Malignant Tumors eTable 3. All-cause Mortality and Short-term Safety Outcomes After Total Knee Replacement Among Continuous Opioid Users vs Opioid-Naive Patients Excluding Patients With Malignant Tumors [file jamanetwopen-2-e198061-s001.pdf]

Supplementary Online Content

Kim SC, Jin Y, Lee YC, et al. Association of preoperative opioid use with mortality and short-term safety outcomes after total knee replacement. *JAMA Netw Open*. 2019;2(7):e198061. doi:10.1001/jamanetworkopen.2019.8061

eTable 1. All-cause Mortality and Short-term Safety Outcomes After Total Knee Replacement Among Intermittent Opioid Users vs Opioid-Naive Patients

eTable 2. Patient Characteristics Prior to Total Knee Replacement Excluding Patients With Malignant Tumors

eTable 3. All-cause Mortality and Short-term Safety Outcomes After Total Knee Replacement Among Continuous Opioid Users vs Opioid-Naive Patients Excluding Patients With Malignant Tumors

This supplementary material has been provided by the authors to give readers additional information about their work.

© 2019 Kim SC et al. *JAMA Network Open.*

eTable 1. All-cause Mortality and Short-term Safety Outcomes After Total Knee Replacement Among Intermittent Opioid Users vs Opioid-Naive Patients

	**Unadjusted model HR (95%CI)**	**Model 1 HR (95%CI)**	**Model 2 HR (95%CI)**
All-cause mortality			
In hospital	1.65 (1.26-2.15)	1.76 (1.35-2.30)	1.20 (0.90-1.59)
30 days	1.39 (1.16-1.67)	1.49 (1.24-1.80)	1.10 (0.90-1.34)
60 days	1.41 (1.19-1.66)	1.52 (1.28-1.80)	1.10 (0.91-1.31)
90 days	1.32 (1.13-1.54)	1.43 (1.22-1.67)	1.02 (0.86-1.21)
Hospital Readmission			
30 days	1.18 (1.13-1.23)	1.21 (1.15-1.26)	0.99 (0.94-1.04)
60 days	1.19 (1.15-1.24)	1.22 (1.18-1.27)	1.00 (0.96-1.04)
90 days	1.20 (1.16-1.24)	1.23 (1.19-1.27)	1.02 (0.99-1.06)
Revision surgery			
30 days	1.60 (1.31-1.96)	1.63 (1.33-2.00)	1.29 (1.04-1.61)
60 days	1.53 (1.30-1.81)	1.56 (1.32-1.84)	1.23 (1.03-1.46)
90 days	1.50 (1.29-1.75)	1.53 (1.31-1.78)	1.23 (1.05-1.45)
Opioid overdose			
30 days	3.88 (1.47-10.20)	3.89 (1.47-10.26)	3.07 (1.12-8.40)
60 days	4.23 (1.62-11.05)	4.30 (1.64-11.26)	3.30 (1.22-8.92)
90 days	4.58 (1.76-11.90)	4.70 (1.80-12.22)	3.48 (1.31-9.26)
Non-vertebral fracture			
30 days	1.82 (0.99-3.36)	1.79 (0.96-3.31)	1.46 (0.76-2.81)
60 days	1.43 (0.99-2.07)	1.43 (0.98-2.07)	1.20 (0.81-1.78)
90 days	1.34 (1.02-1.75)	1.37 (1.04-1.80)	1.16 (0.86-1.55)
Vertebral fracture			
30 days	2.07 (1.44-2.98)	2.15 (1.48-3.14)	1.54 (1.04-2.28)
60 days	1.74 (1.38-2.20)	1.78 (1.40-2.27)	1.33 (1.03-1.71)
90 days	1.80 (1.49-2.16)	1.83 (1.51-2.21)	1.36 (1.11-1.66)
Myocardial infarction or stroke			
30 days	1.14 (0.85-1.51)	1.23 (0.92-1.64)	0.98 (0.72-1.33)
60 days	1.21 (0.94-1.57)	1.32 (1.02-1.71)	1.09 (0.83-1.44)
90 days	1.25 (0.99-1.59)	1.37 (1.08-1.74)	1.10 (0.85-1.42)
Respiratory distress			
30 days	1.86 (1.01-3.42)	1.84 (1.00-3.41)	1.26 (0.66-2.42)
60 days	1.70 (1.02-2.83)	1.70 (1.02-2.85)	1.12 (0.65-1.94)
90 days	1.19 (0.76-1.85)	1.18 (0.75-1.85)	0.79 (0.49-1.28)

© 2019 Kim SC et al. *JAMA Network Open.*

Pneumonia			
30 days	1.13 (0.86-1.50)	1.18 (0.89-1.56)	0.81 (0.60-1.10)
60 days	1.08 (0.85-1.37)	1.12 (0.88-1.43)	0.77 (0.59-1.00)
90 days	1.22 (0.98-1.53)	1.28 (1.02-1.60)	0.86 (0.68-1.09)
Bowel obstruction			
30 days	1.27 (0.89-1.79)	1.32 (0.93-1.87)	1.10 (0.76-1.61)
60 days	1.28 (0.95-1.72)	1.32 (0.98-1.78)	1.10 (0.80-1.51)
90 days	1.32 (1.02-1.72)	1.37 (1.05-1.78)	1.14 (0.86-1.51)

Model 1 is adjusted for age, sex, race/ethnicity and region of residence. Model 2 is adjusted for comorbidity index, frailty, and number of unique prescription drugs in addition to the variables in the Model 1.

© 2019 Kim SC et al. *JAMA Network Open.*

eTable 2. Patient Characteristics Prior to Total Knee Replacement Excluding Patients With Malignant Tumors

	Continuous users (n=19,241)	Intermittent users (n=129,685)	Opioid-naïve (n=107,627)
Presented as percentage or mean ± SD			
Demographics			
Age, years	72.5 ± 5.6	73.5 ± 5.7	74.0 ± 5.8
Female	14,984 (77.9)	94,130 (72.6)	71,688 (66.6)
Race/ethnicity			
White	16,875 (87.7)	114,625 (88.4)	98,262 (91.3)
Black	1,648 (8.6)	7,880 (6.1)	3,948 (3.7)
Hispanic	277 (1.4)	3,007 (2.3)	1,588 (1.5)
Other	271 (1.4)	2,390 (1.8)	2,052 (1.9)
Region			
Northeast	1,978 (10.3)	17,488 (13.5)	19,495 (18.1)
Midwest	4,986 (25.9)	35,562 (27.4)	34,343 (31.9)
South	8,732 (45.4)	52,282 (40.3)	36,021 (33.5)
West	3,539 (18.4)	24,107 (18.6)	17,544 (16.3)
Comorbidities			
Hypertension	17,274 (89.8)	111,792 (86.2)	87,327 (81.1)
Diabetes	7,388 (38.4)	44,010 (33.9)	30,775 (28.6)
Obesity	4,441 (23.1)	25,136 (19.4)	15,088 (14.0)
Back pain	13,680 (71.1)	68,409 (52.8)	37,990 (35.3)
Neuropathic pain	8,591 (44.6)	41,798 (32.2)	19,859 (18.5)
Coronary heart disease	2,155 (11.2)	11,376 (8.8)	6,723 (6.2)
Chronic kidney disease	2,966 (15.4)	15,071 (11.6)	8,395 (7.8)
Heart failure	2,922 (15.2)	12,865 (9.9)	6,629 (6.2)
Hip fracture	89 (0.5)	533 (0.4)	201 (0.2)
Migraine	3,009 (15.6)	14,416 (11.1)	6,825 (6.3)
Sleep disorder	5,227 (27.2)	26,101 (20.1)	14,390 (13.4)
Depression	6,020 (31.3)	23,301 (18.0)	10,912 (10.1)
Anxiety disorder	4,562 (23.7)	17,134 (13.2)	8,217 (7.6)
Bipolar disorder	509 (2.6)	1,714 (1.3)	749 (0.7)
Drug abuse	249 (1.3)	279 (0.2)	50 (0.0)
Alcohol abuse	304 (1.6)	1,228 (0.9)	614 (0.6)
Tobacco use	4,020 (20.9)	17,997 (13.9)	9,611 (8.9)
Rheumatoid arthritis	2,032 (10.6)	7,992 (6.2)	3,558 (3.3)
Frailty			
Robust	2,951 (15.3)	42,041 (32.4)	53,625 (49.8)
Prefrail	12,603 (65.5)	77,858 (60.0)	51,616 (48.0)

© 2019 Kim SC et al. *JAMA Network Open.*

Mild frailty	3,367 (17.5)	9,040 (7.0)	2,272 (2.1)
Moderate-to-severe frailty	320 (1.7)	746 (0.6)	114 (0.1)
Comorbidity index	1.6 ± 2.4	1.1 ± 2.1	0.6 ± 1.7
Medication use			
NSAIDs	9,034 (47.0)	59,123 (45.6)	32,341 (30.0)
Coxibs	2,340 (12.2)	15,054 (11.6)	8,042 (7.5)
Oral corticosteroids	8,816 (45.8)	51,399 (39.6)	29,935 (27.8)
Antidepressants	10,443 (54.3)	43,783 (33.8)	21,700 (20.2)
Benzodiazepines	4,230 (22.0)	15,080 (11.6)	6,600 (6.1)
Bisphosphonates	1,529 (7.9)	9,965 (7.7)	7,222 (6.7)
Anticonvulsants	7,639 (39.7)	25,843 (19.9)	9,460 (8.8)
Health care utilization			
No. of unique prescription drugs	15.4 ± 6.7	12.1 ± 5.6	7.7 ± 4.5
No. of emergency department visit	0.8 ± 1.8	0.5 ± 1.1	0.2 ± 0.6
No. of visits to any physicians	15.7 ± 9.4	13.3 ± 7.6	10.1 ± 6.2
No. of acute hospitalization	0.4 ± 0.2	0.3 ± 0.1	0.1 ± 0.1

© 2019 Kim SC et al. *JAMA Network Open.*

eTable 3. All-cause Mortality and Short-term Safety Outcomes After Total Knee Replacement Among Continuous Opioid Users vs Opioid-Naive Patients Excluding Patients With Malignant Tumors

	**Unadjusted HR (95% CI)**	**Model 1 HR (95% CI)**	**Model 2 HR (95%CI)**
All-cause mortality			
In hospital	1.61 (0.97, 2.68)	**1.95 (1.17, 3.25)**	0.98 (0.56, 1.69)
30 days	1.33 (0.91, 1.95)	**1.65 (1.12, 2.42)**	0.95 (0.63, 1.43)
60 days	**1.56 (1.12, 2.16)**	**1.95 (1.40, 2.71)**	1.08 (0.76, 1.54)
90 days	**1.45 (1.06, 1.98)**	**1.81 (1.32, 2.48)**	1.00 (0.71, 1.40)
Hospital Readmission			
30 days	**1.48 (1.36, 1.62)**	**1.57 (1.44, 1.72)**	1.05 (0.96, 1.16)
60 days	**1.58 (1.47, 1.69)**	**1.67 (1.55, 1.79)**	**1.12 (1.03, 1.21)**
90 days	**1.62 (1.52, 1.73)**	**1.71 (1.61, 1.83)**	**1.17 (1.10, 1.26)**
Revision surgery			
30 days	**2.72 (1.96, 3.77)**	**2.74 (1.96, 3.83)**	**1.66 (1.15, 2.40)**
60 days	**2.24 (1.69, 2.95)**	**2.32 (1.75, 3.07)**	**1.38 (1.01, 1.89)**
90 days	**2.31 (1.79, 2.98)**	**2.38 (1.84, 3.09)**	**1.49 (1.12, 1.98)**
Opioid overdose			
30 days	**7.36 (2.25, 24.13)**	**7.04 (2.12, 23.39)**	3.65 (0.98, 13.66)
60 days	**11.16 (3.74, 33.31)**	**10.99 (3.64, 33.23)**	**5.66 (1.68, 19.05)**
90 days	**18.83 (6.84, 51.82)**	**19.41 (6.98, 54.01)**	**9.60 (3.16, 29.15)**
Non-vertebral fracture			
30 days	**3.21 (1.20, 8.55)**	**3.36 (1.25, 9.03)**	2.25 (0.75, 6.70)
60 days	1.67 (0.84, 3.35)	1.67 (0.83, 3.36)	1.13 (0.53, 2.42)
90 days	**2.03 (1.26, 3.28)**	**2.15 (1.33, 3.50)**	1.49 (0.88, 2.53)
Vertebral fracture			
30 days	**4.25 (2.51, 7.20)**	**4.54 (2.65, 7.78)**	**2.32 (1.28, 4.21)**
60 days	**4.29 (3.05, 6.02)**	**4.58 (3.24, 6.48)**	**2.44 (1.66, 3.60)**
90 days	**4.53 (3.45, 5.94)**	**4.77 (3.62, 6.29)**	**2.47 (1.82, 3.36)**
Myocardial infarction or stroke			
30 days	0.84 (0.42, 1.69)	1.04 (0.52, 2.10)	0.72 (0.34, 1.50)
60 days	0.94 (0.51, 1.72)	1.17 (0.63, 2.15)	0.85 (0.45, 1.62)

© 2019 Kim SC et al. *JAMA Network Open.*

90 days	1.14 (0.68, 1.91)	1.43 (0.85, 2.40)	0.93 (0.54, 1.62)
Respiratory distress			
30 days	2.06 (0.66, 6.38)	2.13 (0.68, 6.68)	1.30 (0.38, 4.46)
60 days	**2.59 (1.07, 6.24)**	**2.61 (1.07, 6.37)**	1.23 (0.46, 3.27)
90 days	**2.44 (1.18, 5.07)**	**2.48 (1.18, 5.20)**	1.20 (0.52, 2.72)
Pneumonia			
30 days	**1.80 (1.09, 2.95)**	**1.99 (1.21, 3.29)**	0.98 (0.56, 1.71)
60 days	**1.74 (1.13, 2.69)**	**1.90 (1.23, 2.96)**	0.87 (0.54, 1.43)
90 days	**2.10 (1.43, 3.07)**	**2.30 (1.56, 3.38)**	1.01 (0.65, 1.55)
Bowel obstruction			
30 days	**2.56 (1.40, 4.66)**	**2.86 (1.56, 5.27)**	**1.97 (1.01, 3.85)**
60 days	**2.58 (1.55, 4.28)**	**2.87 (1.71, 4.80)**	**2.01 (1.14, 3.53)**
90 days	**2.30 (1.45, 3.67)**	**2.54 (1.58, 4.08)**	**1.79 (1.07, 3.01)**

Model 1 is adjusted for age, sex, race/ethnicity and region of residence. Model 2 is adjusted for comorbidity index, frailty, and number of unique prescription drugs in addition to the variables in the Model 1.

© 2019 Kim SC et al. *JAMA Network Open.*
